# Ureteral urothelial carcinoma with squamous cell carcinoma and sarcomatoid carcinoma differentiation: a case report

**DOI:** 10.1186/s12893-021-01099-1

**Published:** 2021-02-21

**Authors:** Shangqing Ren, Hualin Feng, Yige Bao, Yi Wei, Yong Ou, Yaoqian Wang, Qian Lv, Shan Zhong, Fang Zhou, Shida Fan, Qiang Wang, Cheng Luo, Zhengjun Chen, Yu Nie, Dong Wang

**Affiliations:** 1 Department of Robotic Minimally Invasive Surgery Center, Sichuan Academy of Medical Sciences & Sichuan Provincial People’s Hospital, No. 32 West Second Section First Ring Road, Chengdu, Sichuan China; 2 Departments of Urology, Institute of Urology, West China Hospital, Sichuan University, Chengdu, Sichuan China; 3 Department of Organ Transplantation Center, Sichuan Academy of Medical Sciences & Sichuan Provincial People’s Hospital, Chengdu, Sichuan China

**Keywords:** Upper urinary tract urothelial carcinoma, Squamous cell carcinoma, Sarcomatoid carcinoma, Case report

## Abstract

**Background:**

Upper urinary tract urothelial carcinoma (UTUC) with multiple pathological types is extremely rare in the clinic, but the recurrence rate and mortality these patients are high. At present, there is no standard treatment for such cases.

**Case presentation:**

We reported a case of ureteral urothelial carcinoma with squamous cell carcinoma and sarcomatoid carcinoma differentiation and rapid ileal metastasis and reviewed the literature related to different pathological types of upper urinary tract tumours to explore the diagnosis, treatment and prognosis characteristics of the disease, enhance our understanding of its clinical manifestations and history of evolution and provide guidance for avoiding missed diagnosis and misdiagnosis**.**

**Conclusion:**

There is no standard treatment for urinary malignant tumours with multiple pathological types; radical surgery is considered a suitable choice. Chemotherapy, targeted drug therapy and immunotherapy may be beneficial to the survival of patients. In short, these patients have a high risk of recurrence and metastasis and a poor prognosis.

## Background

Upper urothelial carcinoma (UTUC) is a rare tumour, accounting for 5–7% of all kidney tumours and 5–10% of all urothelial tumours [1], with an annual incidence of 1–2/100,000 in Western populations [2]. Tobacco and aromatic amine exposure are the most common risk factors for UTUC, and smoking increases the risk of disease by 2.5- to 7-fold [2]. The most common symptom of UTUC is haematuria, followed by ipsilateral abdominal pain, in addition, systemic symptoms include anorexia, weight loss, fatigue, fever, etc. [2].The clinical manifestations of such diseases are often nonspecific, so patients are prone to missed diagnosis and misdiagnosis, and the pathological stage is usually late once they are detected. Therefore, the overall prognosis of these patients is poor. The postoperative pathological diagnosis of the patient in this case was high-grade urothelial carcinoma of the ureter with squamous cell carcinoma and sarcomatoid carcinoma differentiation, and ileal metastasis occurred within one year after the operation. This case is extremely rare; at present, there are no similar cases in the literature at home or abroad.

## Case presentation

**Fig. 1:**
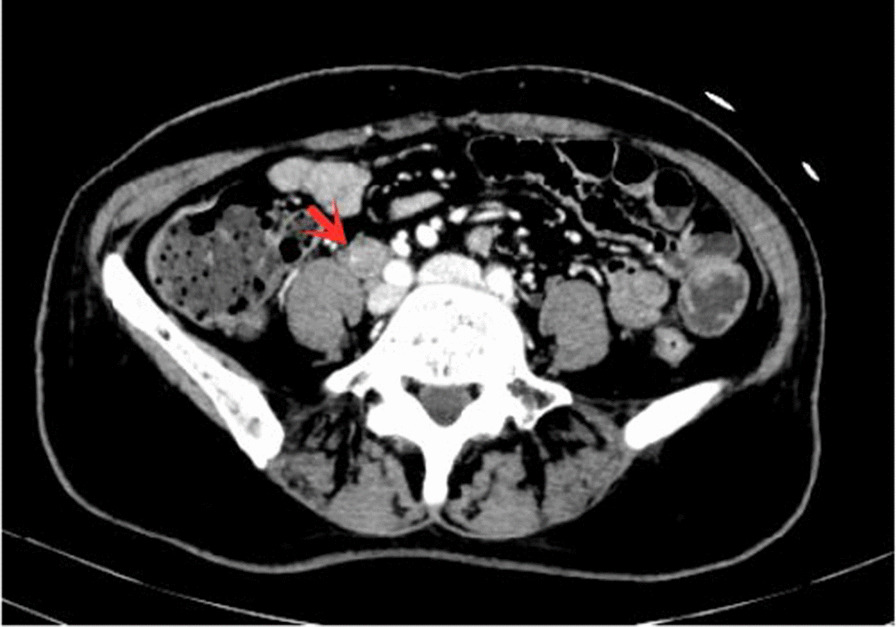
The wall of the middle section of the right ureter is obviously thickened, with stenosis, occlusion, and local nodular changes observed

**Fig. 2:**
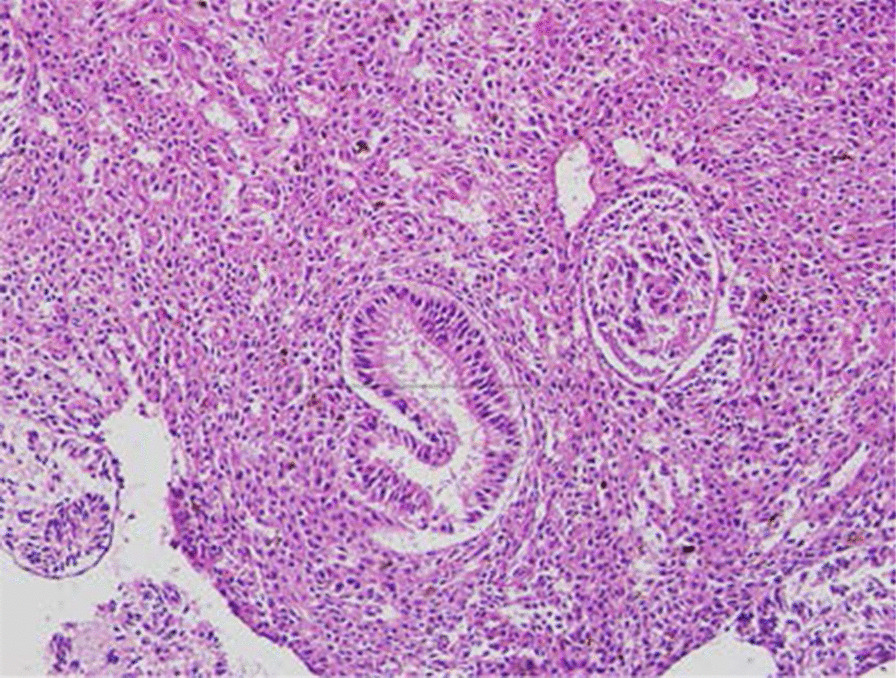
High-grade urothelial carcinoma of the right ureter with differentiation of squamous cell carcinoma and sarcomatoid carcinoma

**Fig. 3:**
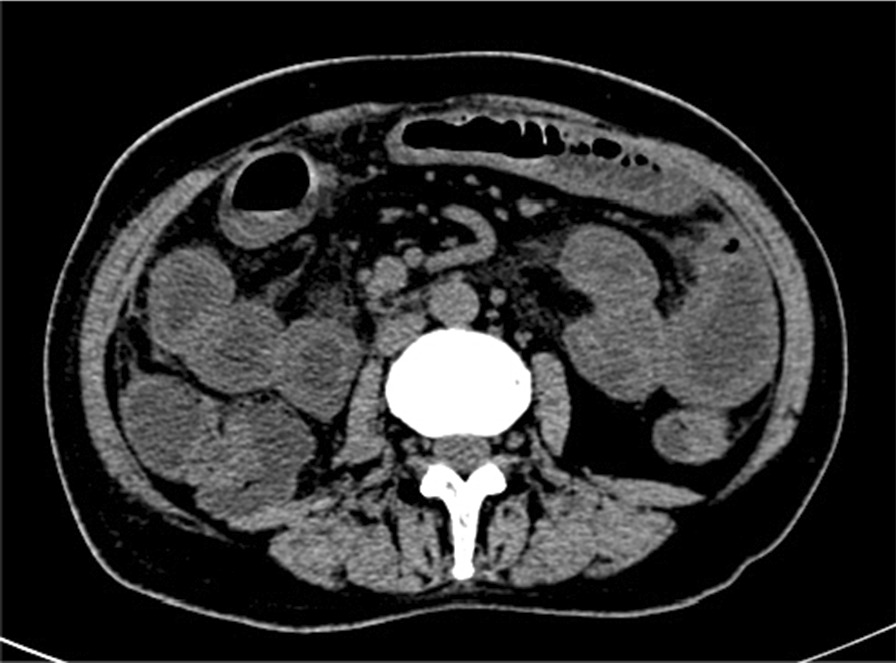
A cystic shadow can be seen on the right side of the pelvis and the rectum and uterus, with blurred edges and unclear boundaries from the adjacent bowel

**Fig. 4:**
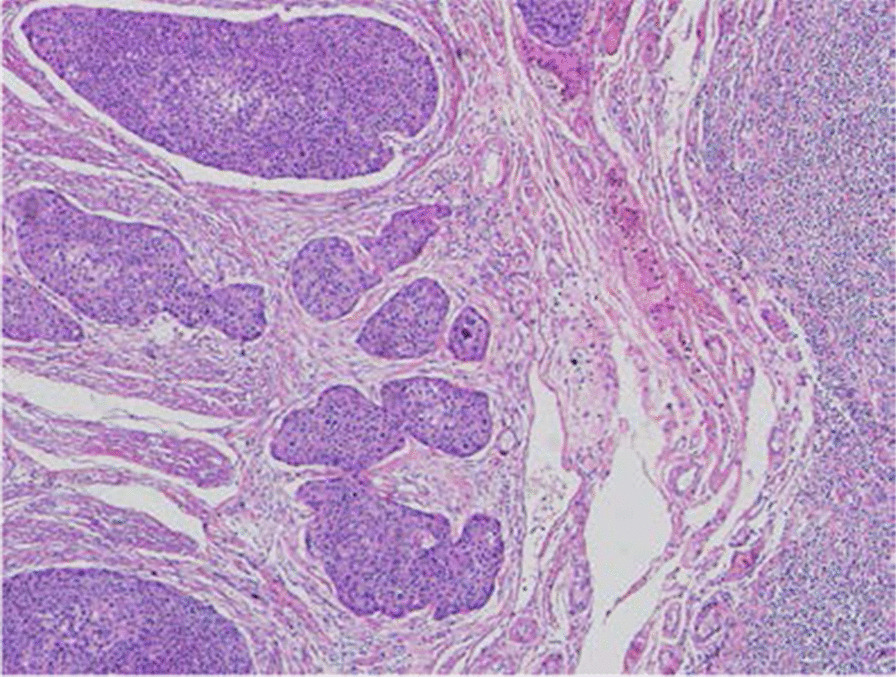
Diagnosis of secondary urothelial carcinoma in pathological specimens of the ileum

**Fig. 5:**
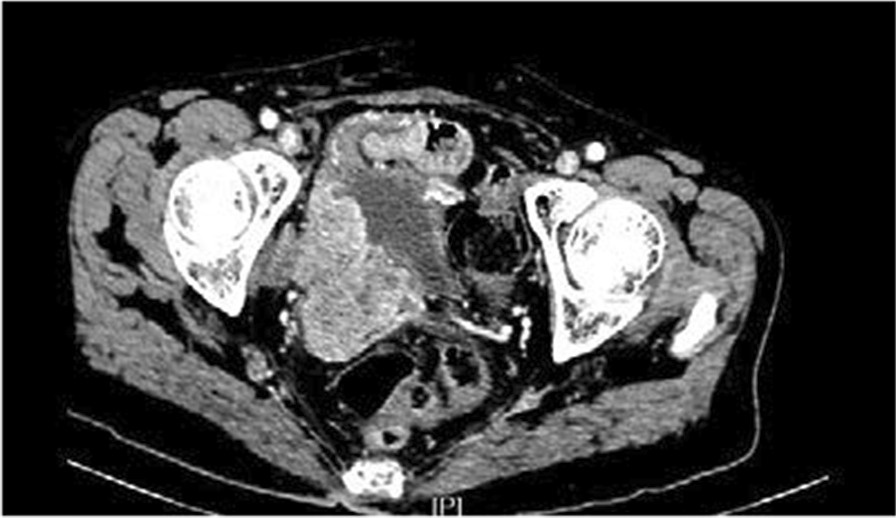
Irregular soft tissue mass shadows on the right side of the pelvis. Tumour metastasis with invasion of the inferior vena cava and right common iliac artery was considered

**Fig. 6:**
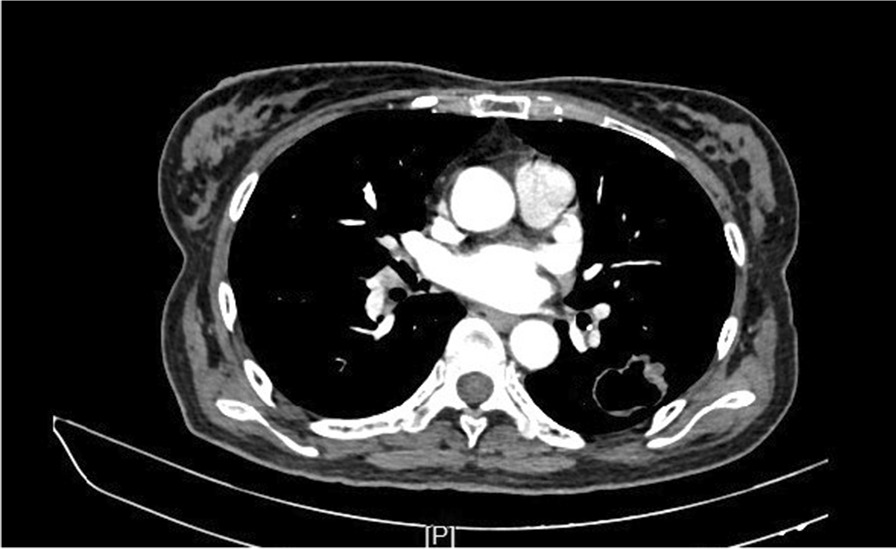
There is a mass shadow in the dorsal segment of the left lower lobe, approximately 35 × 24 × 32 mm in size, with lobes, cavities, uneven wall thickness, and enhancement

**Fig. 7:**
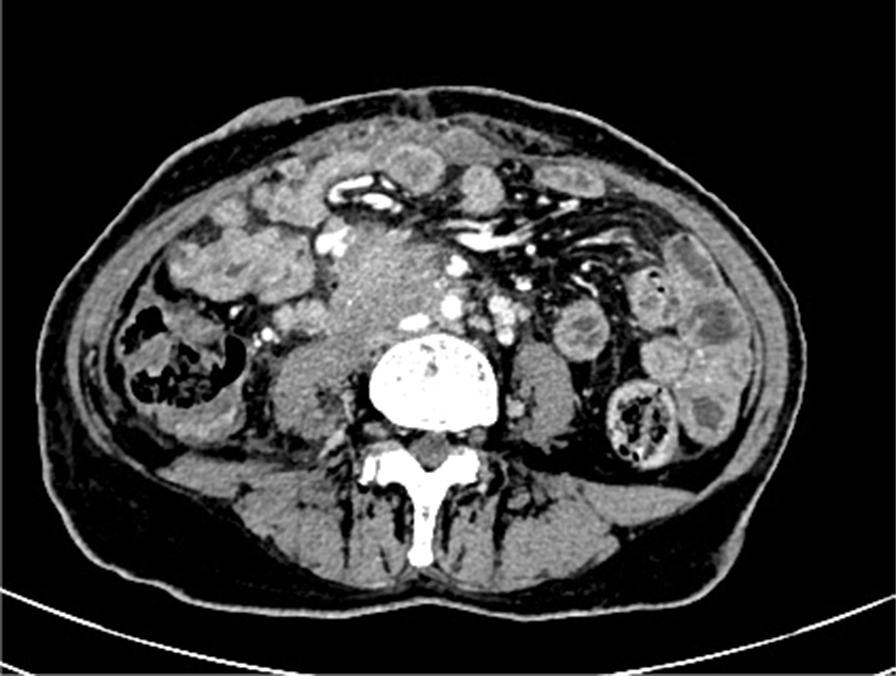
Tumour invades and wraps right iliac blood vessel

**Fig. 8:**
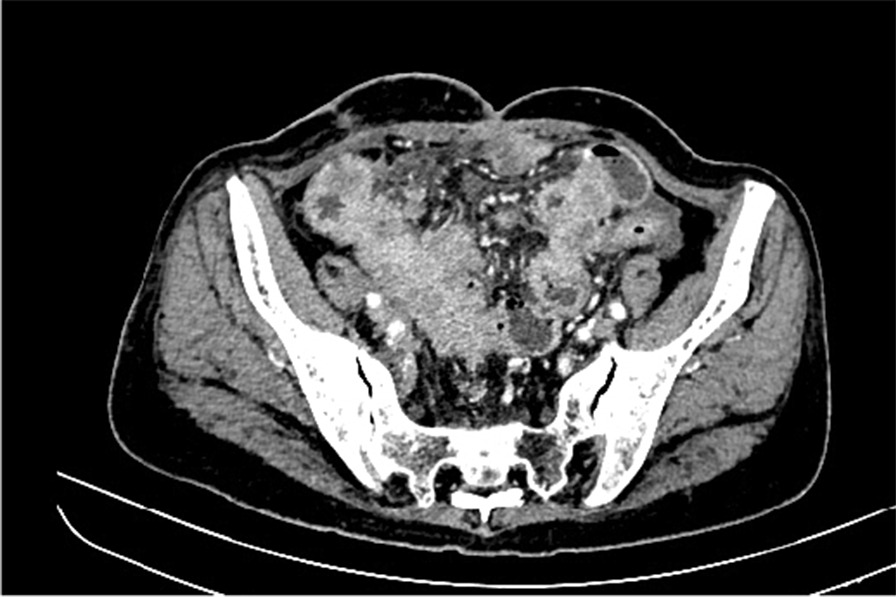
Retroperitoneal soft tissue density shadows, multiple retroperitoneal lymph nodes, pelvic, intestinal, and bladder lesions, and metastases were considered

A 63-year-old female patient was admitted to the hospital in January 2019 after physical examination revealed "space occupying the right ureter with hydronephrosis in the right kidney for one month". Colour Doppler ultrasound revealed a 5.0 cm × 1.5 cm hypoechoic region was noted in the abdominal segment of the right ureter. CT revealed (Fig. 1): the middle ureteral wall of the right side was thickened with stenosis and occlusion, with local nodular changes of approximately 4.5 cm in length. Cystoscopy of the urethra revealed no obvious abnormalities in the bladder and urethra. Upon urine exfoliative cytology examination, nuclear atypical cells were found. Accompanying transurethral right ureteroscopy biopsy revealed urothelial carcinoma. One week later, transabdominal robot-assisted laparoscopic right nephrectomy + right ureterectomy + bladder wall cuff resection was performed. Postoperative pathological diagnosis (Fig. 2) was high-grade urothelial carcinoma of the right ureter with squamous cell carcinoma and sarcomatoid carcinoma differentiation. The tumour infiltrated into the serosa of the ureteral wall, but the short end of the ureter, kidney and perirenal fat tissue were not involved. For a half a year after the operation, the patient was followed up regularly every 3 months by cystoscopy, and bladder perfusion was administered for symptomatic treatment to prevent bladder recurrence. No other abnormalities were noted during follow-up. Due to the COVID-19 epidemic, follow-up was interrupted for 8 months. Fifteen months after the operation, she was readmitted to the hospital for "abdominal pain, abdominal distension with no flatulence or defecation for 1 week". CT (Fig. 3) revealed a cystic shadow of the right pelvis can be seen in the lateral and rectal uterine depression, with blurred edges and unclear boundaries from the adjacent bowel. Tumour indicators CA125 and CA19-9 were 71.8 U/ml and 37.23 U/ml, respectively. The patient had obvious symptoms of gastrointestinal obstruction and required active surgical exploratory decompression treatment, Ileectomy + side-to-side anastomosis + release of intestinal adhesions + bowel decompression was performed. During the operation, a solid mass of 5 cm × 5 cm on the right posterior wall of the right pelvic cavity was fixed in the pelvic wall, and the small intestine were close to the bladder and 25 cm from the ileocecal part. The proximal obstructed small intestine was dilated, the mass was suspected of tumour recurrence. After communicating with the patient about their condition, the patient’s family requested that resection not be considered at that time. The pathological diagnosis (Fig. 4) was as follows: "ileum": consistent with secondary urothelial carcinoma. The maximum diameter of the tumour was approximately 4.5 cm, and it infiltrated the whole layer of the intestinal wall. There was no tumour invasion at the incisal margin, but tumour metastasis was noted in the peri-intestinal lymph nodes around the intestine (1/2). Molecular examination revealed no FGFR alteration was detected on the pathological slides, so she can't participate in clinical trials of targeted drug Erdafitinib in hospitals. Follow-up of CT showed (Figs. 5, 6): a patchy soft tissue shadow was observed adjacent to the right common iliac artery, and an irregular soft tissue mass was noted on the right side of the pelvis. The dorsal segment of the left lower lobe and the posterior segment of the right upper lobe were considered for lung metastasis. The patient was enrolled in a phase III clinical trial for the treatment of locally advanced or metastatic urothelial cancer, and tislelizumab immunotherapy combined with carboplatin + gemcitabine chemotherapy was administered every 3 weeks for a total of 4 cycles, but the effect was not optimal. Re-examination of chest and abdomen CT 20 at months after surgery (Figs. 7, 8) revealed multiple groups of lymph node metastases in the retroperitoneum and in multiple organs, such as the liver, intestine, bladder, lumbar spine, pelvis, and left lung. She died of multiple organ failure 19 months after surgery.

## Discussion

UTUC is similar in type and morphology to bladder cancer and can be divided into non-invasive papillary tumours, low malignant potential tumours, low or high papillary urothelial tumours, carcinoma in situ and invasive carcinoma. Squamous cell carcinoma of the upper urinary tract accounts for less than 10% of renal pelvic tumours and is even rarer in the ureter. Other variants include micropapillary carcinoma and sarcomatoid carcinoma and lymphoepithelioma [3]. In the multidisciplinary comprehensive treatment of UTUC, RNU and cyst sleeve resection [4–6] are the current gold standards of treatment. For suitable patients with low risk, some scholars have recommended kidney preservation surgery, combined with BCG or mitomycin-based intracavitary chemical and immunotherapy [7]. Regarding chemotherapy for UTUC, a meta-analysis with a sample size of 328 patients showed that [8] neoadjuvant chemotherapy before RNU may improve the survival outcomes of patients with locally advanced UTUC. In overall survival (OS), both tumour-specific survival (CSS) and progression-free survival (PFS) showed absolute increases, which were 11%, 18%, and 13%, respectively. Patients with high-risk or locally advanced UTUC who receive cisplatin-based adjuvant chemotherapy have significant benefits in overall survival and disease-free survival compared with RNU alone [9].Therefore, the use of NAC or postoperative adjuvant chemotherapy for UTUC patients with multiple pathological types may be appropriate, which is worthy of further study. Radiation therapy may be of little significance to such patients.

Squamous cell carcinoma of the renal pelvis and ureter is a relatively rare malignant tumour of the urinary system, and its mechanism is closely related to chronic stimulation, chronic infection and chronic inflammation [10]. However, there are no risk factors for long-term chronic stimulation in this case. Due to the insidious onset of squamous cell carcinoma of the renal pelvis and ureter, diagnosis is difficult, and most patients are already in a late stage when diagnosed. It has been reported that renal fine needle aspiration cytology (FNAC) can be used as a tool to diagnose squamous cell carcinoma of the renal collecting system [11], and 8F-fluorodeoxyglucose-positron emission tomography/computed tomography (18F- FDG-PET/CT) can detect metastasis early and improve survival [12]. Most of the reports on such diseases are case reports, and there is a lack of systematic large sample studies, so there is no standard treatment at present. Most patients choose radical nephroureterectomy and bladder cuff resection as the main surgical treatment. Some scholars have proposed that platinum-based combined chemotherapy may benefit patients [13]. Radiotherapy is rarely used in current treatments, and there are no reports on the application of molecular targeted drug therapy or immunotherapy to the disease. It has been reported that if the pathological type of UTUC contains squamous differentiation [14], it is more likely to have invasive tumour characteristics, and the postoperative prognosis is poor; the 5-year specific survival rate is 48.9%, and the median survival time is 59 months.

Sarcomatoid carcinoma of the renal pelvis and ureter is another rare malignant tumour with a mixed phenotype, accounting for approximately 7% of histological variation of renal pelvis and ureteral malignant tumours [15]. Its biological characteristics are also invasive. Even if patients are treated actively, the survival rate is still low. Some studies have mentioned that previous cyclophosphamide treatment or radiotherapy may lead to the evolution of urothelial cancer to sarcomatoid cancer [16]. According to the classification of urological tumours in the 2016 edition of the WHO, all urothelial carcinomas containing sarcomas are classified as sarcomatous changes of uroepithelial carcinoma [17], but sarcomatoid carcinomas and carcinosarcomas are different in terms of the cell of origin. At present, most of the patients reported choosing to undergo radical surgery, but some studies have suggested that adjuvant radiotherapy and chemotherapy may improve patient prognosis. However, there is a lack of randomized controlled trials for further confirmation [16], and there are no reports about molecular targeted drug therapy and immunotherapy for sarcomatoid carcinoma of the renal pelvis and ureter. Compared with patients with urothelial cancer only, patients with sarcomatoid cancer are usually diagnosed at a later stage and have a worse prognosis [18]. Few cases survive for more than two years [19].

This case of UTUC had both squamous cell carcinoma and sarcomatoid carcinoma, and ileal infiltration and metastasis occurred. It was clinically staged as localized tumor by using preoperative imaging assessment, surgery for this patient followed a transperitoneal approach, the postoperative pathology suggested T3. Although non-tumor principle was strictly followed, it did not rule out the possibility of dissemination occurred during the surgery in this stage. Considering that recurrence, metastasis and late clinical staging are related to the pathological characteristics of the tumour, combined with the subjective demands of the patient and family members, after gastrointestinal symptoms appeared, palliative treatment was chosen instead of a series of surgical treatments, such as the more traumatic pubic resection.While It was uncertain that the recurrence was related to the choice of surgical method, the pathological result was rare, and the preoperative diagnosis and staging were so difficult, we still hope that this case report will give a reminder to consider open surgery to achieve better curative effects for similar cases in after years.

At present, the treatment of UTUC has benefitted from the discovery of promising biomarkers and therapeutic targets in genetics and molecular biology, increasing the possibility that molecular targeted drug therapy or immunotherapy could be incorporated into UTUC treatment approaches. Molecular targeted drug therapy or immunotherapy is mainly used in patients with advanced or metastatic urothelial cancer. Some studies [20] have found that T cell depletion is one of the main phenotypes of genetic changes in patients with UTUC. FGFR3 may be a regulator of the UTUC immune environment by weakening interferon γ (IFNG) signalling, and it is suggested that interferon response genes may be upregulated by inhibiting FGFR3 or PD-1/PD-L1, thereby reversing T cell depletion in UTUC and remodelling the immune environment, which provides a theoretical basis for targeted therapy. One clinical trial [21] has shown that the FGFR inhibitor erdafitinib has offered significant benefit in the treatment of advanced urothelial cancer patients with FGFR changes, with a remission rate of approximately 40% (complete remission rate 3%, partial remission rate 37%). However, in this case, the patient's genetic test was negative for FGFR, so relevant molecular targeting drugs could not be used. Programmed death receptor 1 (PD-1) and its ligand (PD-L1) are important negative regulators of immune activity that can prevent the destruction of normal tissue and autoimmunity. PD-1/PD-L1 drugs approved by the FDA for the treatment of urothelial cancer are passing various stages of clinical trials with good objective response rates and controllable adverse reactions [7, 22]. In addition, some studies have found that the expression of PD-1 and PD-L1 in patients with UTUC accompanied by squamous cell differentiation is significantly higher than that in patients with simple UTUC [23]. Perhaps immunotherapy will bring hope to these patients. In the future, molecular targeted drugs and immunotherapy may become a first-line strategy for the treatment of upper urinary tract urothelial cancer and multipathological types of ureteropelvic malignant tumours, and precision medicine could improve the prognosis of such patients.

## Conclusion

In summary, upper urinary tract urothelial carcinoma exhibits a variety of differentiation types and considerable variation. In this case, renal pelvis and ureteral urothelial carcinoma with squamous cell carcinoma and sarcomatoid carcinoma differentiation and ileal metastasis showed rapid progression, a high degree of malignancy, a high risk of metastasis and a poor prognosis. Colour Doppler ultrasound, CT, PET/CT, MRU and urine exfoliative cytology are good auxiliary examinations for the diagnosis of this disease, which mainly depends on postoperative pathological biopsy and immunohistochemistry. At present, there is no standard treatment for this kind of case, and radical surgery is considered a more suitable choice. Chemotherapy, targeted drug therapy and immunotherapy may be beneficial to patient survival. In short, urinary malignant tumours with multiple pathological types have a high risk of recurrence and metastasis and a poor prognosis. Further research is needed to understand their natural medical history and prognostic factors. Early diagnosis and early treatment are the keys to improving the survival rate. At the same time, regular postoperative follow-up is recommended.

## Data Availability

All the data and material are from the patient’s assay and examination of Sichuan Academy of Medical Sciences & Sichuan Provincial People’s Hospital, which are real, credible and available.

## Abbreviations

UTUC: Upper urinary tract urothelial carcinoma
UCB: Urothelial carcinoma of the bladder
CT: Computerized tomography
FGFR: Fibroblast growth factor receptor
MRI: Magnetic Resonance Imaging
FNAC: Fine needle aspiration cytology
18F-FDG-PET/CT: 18F-fluorodeoxyglucose-positron emission tomography/computed tomography
WHO: World Health Organization
EMA: Epithelial membrane antigen
RNU: Radical nephroureterectomy
BCG: Bacillus Calmette-Guerin
OS: Overall survival
CSS: Carcinoma-specific survival
PFS: Progression-free survival
NAC: Neo Adjuvant Chemotherapy
IFNG: Interferon Gamma
PD-1: Programmed cell death 1
PD-L1: Programmed cell death-Ligand 1
FDA: Food and drug administration
MRU: Magnetic resonance urography
